# Subtype-dependent difference of glucose transporter 1 and hexokinase II expression in craniopharyngioma: an immunohistochemical study

**DOI:** 10.1038/s41598-020-80259-4

**Published:** 2021-01-08

**Authors:** Naoto Mukada, Masahiko Tosaka, Nozomi Matsumura, Rei Yamaguchi, Masanori Aihara, Koji Isoda, Tetsuya Higuchi, Yoshito Tsushima, Hideaki Yokoo, Yuhei Yoshimoto

**Affiliations:** 1 Department of Neurosurgery, Gunma University Graduate School of Medicine, 3-39-22 Showa-machi, Maebashi, Gunma 371-8511 Japan; 2 Department of Human Pathology, Gunma University Graduate School of Medicine, Maebashi, Gunma Japan; 3 Department of Diagnostic Radiology and Nuclear Medicine, Gunma University Graduate School of Medicine, Maebashi, Gunma Japan

**Keywords:** Cancer, Endocrinology, Oncology

## Abstract

Papillary craniopharyngiomas are characterized by the *BRAF* V600E mutation. Enhancement of glucose metabolism may be involved in the downstream of the *BRAF* V600E mutation in many types of tumors. Glucose metabolism was investigated in craniopharyngioma using immunohistochemical analysis. The study included 29 cases of craniopharyngioma (18 adamantinomatous type [ACP], 11 papillary type [PCP]). Immunohistochemical analysis was performed with anti-glucose transporter-1 (GLUT-1), anti-hexokinase-II (HK-II), anti-BRAF V600E, and anti-beta-catenin antibodies. Expressions of GLUT-1 and HK-II were evaluated using a semiquantitative 4-tiered scale as 0, 1+, 2+, 3+, and divided into negative (0 or 1+) or positive (2+ or 3+) group. GLUT-1 expression level was significantly higher in PCPs than ACPs (0, 1+, 2+, 3+ = 2, 12, 4, 0 cases in ACP, respectively, 0, 1+, 2+, 3+ = 0, 2, 5, 4 in PCP, p = 0.001), and most PCPs were classified into positive group (positive rate, 22.2% [4/18] in ACP, 81.8% [9/11] in PCP; p = 0.003). HK-II expression was also conspicuous in PCPs (0, 1+, 2+, 3+ = 7, 9, 2, 0 cases in ACP, 0, 3, 3, 5 in PCP; p = 0.001), and most of them divided into positive group (positive rate, 11.1% [2/18] in ACP, 72.7% [8/11] in PCP; p = 0.001). Expression patterns of BRAF V600E and beta-catenin reflected the clinicopathological subtypes. Both GLUT-1 and HK-II expressions were prominent in PCP. Glucose metabolism might be more enhanced in PCP than ACP. PCP may use the glucose metabolic system downstream of the BRAF V600E mutant protein.

## Introduction

**Figure 1:**
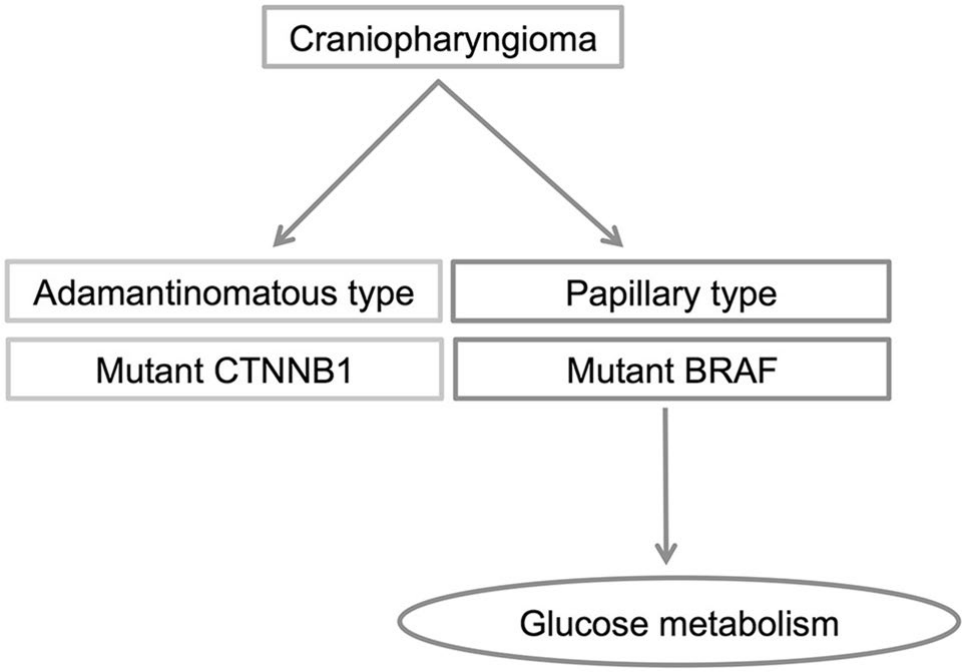
Relationship between clinicopathological subtypes in craniopharyngioma and enhancement of glucose metabolism, including immunohistochemical overexpression of GLUT-1, HK-II, and high uptake of [^18^F]fluorodeoxyglucose.

Craniopharyngioma is a sellar and/or suprasellar skull base tumor, which is surrounded by critical structures, and consequently is difficult to remove completely and recurs repeatedly. Craniopharyngiomas can be classified into two histological subtypes, adamantinomatous craniopharyngiomas (ACPs) and papillary craniopharyngiomas (PCPs). ACPs occur in both children and adults, whereas PCPs mainly occur in adults. ACP occurs as a lobulated tumor with cyst formation, which contains a machine oil-like dark brown fluid. Histologically, ACPs have epithelium arranging whorls, cords, lobules, and basal palisading, stellate reticulum, and “wet” keratin are often detected^1^. PCPs have well-differentiated monomorphic squamous epithelium, with fibrovascular cores, thin capillary blood vessels, and scattered immune cells^1^. Recently, the *BRAF* V600E mutation was identified in 94.4% of PCPs but in 0% of ACPs. The *CTNNB1* mutation in the exon 3 degradation-targeting motif was detected in 96% of ACPs and 0% of PCPs^2^. These changes have been established as the driver mutations in several neoplasms. These two gene alterations are mutually exclusive in the ACP and PCP, and so are useful as diagnostic molecular markers (Fig. 1)^2,3^.

Colorectal cancer cells with *KRAS* or *BRAF* mutations show enhanced glucose metabolism such as glucose uptake and glycolysis, and require expression of glucose transporter-1 (GLUT-1), a membrane glucose transporter^4,5^. GLUT-1 expression is elevated in papillary thyroid carcinomas with *BRAF* mutations^6–8^. Colorectal cancers with *BRAF* mutations have higher glucose uptake on [^18^F]fluorodeoxyglucose positron emission tomography (FDG-PET) than those without *BRAF* mutations^5^. Glucose metabolism is reported to be enhanced in many tumors with *BRAF* mutations (Fig. 1).

This immunohistochemical study examined the expression of glycometabolism-related enzymes in the two clinicopathological variants of craniopharyngioma.

## Methods

### Patients

A total of 41 craniopharyngioma cases removed at Gunma University Hospital from August 2000 to March 2019 were collected. We excluded 7 cases due to a few tumor tissues with marked xanthogranulomatous change. Five of the other 34 cases were recurrent after gamma knife treatment and were excluded because of irregular histopathological findings. Therefore, 29 cases including 15 men and 14 women (median age, 44 years, interquartile range [IQR], 38–58) were utilized in this study. This study was reviewed and approved by the institutional review board of Gunma university graduate school of medicine.

### Immunohistochemical study

All specimens were fixed with 10% formalin and embedded in a paraffin block. Immunohistochemical examination was done on paraffin-embedded sections cutting at a thickness of 2.5 μm using the biotin-streptavidin immunoperoxidase method (Histofine Kit, Nichirei, Tokyo, Japan). Antibodies against following antigens were used: GLUT-1 (monoclonal, 1: 100, Abcam, Cambridge Science Park, Milton, UK), hexokinase-II (HK-II) (monoclonal, 1: 200, Abcam, Cambridge Science Park, Milton, UK), BRAF V600E (clone VE1, monoclonal, 1: 50, Spring Bioscience, Pleasanton, CA, USA), and beta-catenin (monoclonal, 1: 100, BD Transduction Laboratories, Tokyo, Japan). For antigen retrieval, sections for BRAF V600E were heated by a microwave in 0.01-mol/L citrate buffer (pH 6.0) for 15 min at 98 °C, and sections for GLUT-1, HK-II and beta-catenin were autoclaved in 0.01-mol/L citrate buffer (pH 6.0) for 10 min at 121 °C before staining. After visualization with diaminobenzidine, sections were briefly counterstained with hematoxylin^9^.

The intensity, expression pattern of staining, and percentage of positive cells were assessed for each specimen and the results of GLUT-1 and HK-II immunostating were evaluated using a following semiquantitative 4-tiered scale; 0: 0%, 1+: 0–10%, 2+: 11–50%, and 3+: 51–100% as described previously^9^. Based on the results, the cases were classified into the negative (0 or 1+) or positive (2+ or 3+) group.

### FDG-PET

FDG-PET imaging before surgery was carried out in 7 of 29 cases (24.1%). Image findings and maximum standardized uptake value (SUVmax) were evaluated in 7 cases. FDG-PET imaging was performed with a dedicated scanner as described previously^10^. Image acquisition was initiated 50 min after injection of 5–6 MBq/kg of FDG after more than 6 h fasting. Three-dimensional data acquisition was performed for 3 min per bed position, followed by imaging reconstruction with the three-dimensional ordered subset expectation maximization method. Correction of segmented attenuation was performed by computed tomography (CT) radiography (140 kV, 120–240 mA) to produce 128 × 128 matrix images. SUV was calculated as follows: radioactive concentration in the region of interest (MBq/g)/injected dose (MBq)/patient body weight. Region of interest analysis was conducted by a nuclear physician with the aid of the corresponding CT scans. Only FDG-PET data partially overlaps with the previous report^10^.

### Statistical analysis

All values were reported as median (IQR). Between group comparisons were performed using the Mann–Whitney test. The Fisher exact probability test was used in 2 × 2 tables. p values of < 0.05 were considered to indicate significant difference.

### Ethical approval

This study was conducted under the institutional review board approval. The institutional review boards approved an opt-out method of informed consent. All procedures performed in studies involving human participants were in accordance with the ethical standards of the institutional and/or national research committee and with the 1964 Helsinki declaration and its later amendments or comparable ethical standards.

## Results

### Clinical and imaging characteristics

The patient characteristics are presented in Table 1. Of the 29 patients with craniopharyngioma, 18 (62%) patients had histological diagnoses of ACP, and 11 (38%) of PCP. Calcification was observed on CT only in 17 cases of ACP, and no cases in PCP (positive rate, 94.4% [17/18] in ACP, 0% [0/11] in PCP; p < 0.01). Other clinical and imaging characteristics did not differ between ACP and PCP.

**Table 1:** Clinical and imaging characteristics, and immunostaining findings.

	Total, n = 29 (%)	ACP, n = 18 (%)	PCP, n = 11 (%)	p value
**Age**				
0–19	4 (13.8)	4 (22.2)	0 (0.0)	
20–39	6 (20.7)	4 (22.2)	2 (18.2)	
40–59	12 (41.4)	8 (44.4)	4 (36.4)	
60–79	7 (24.1)	2 (11.1)	5 (45.5)	
**Sex**				
M	15 (51.7)	7 (38.9)	8 (72.7)	0.128
F	14 (48.3)	11 (61.1)	3 (27.3)	
**Size**				
Median	34.8	37.5	28.4	0.105
IQR	23.6–41	27.1–44.7	22.9–37.3	
**Cyst formation**				
+	17 (58.6)	13 (72.2)	4 (36.4)	0.13
−	12 (41.4)	5 (27.8)	7 (63.6)	
**Calcification**				
+	17 (58.6)	17 (94.4)	0 (0.0)	< 0.01
−	12 (41.4)	1 (5.6)	11 (100)	
**Approach**				
Nasal	19 (65.5)	12 (66.7)	7 (63.6)	1
Craniotomy	10 (34.5)	6 (33.3)	4 (36.4)	
**Past surgery**				
Initial surgery	26 (89.7)	16 (88.9)	10 (90.9)	1
Recurrent tumor	3 (10.3)	2 (11.1)	1 (9.1)	
**Removal extent**				
Median	95	95	95	0.98
IQR	90–100	90–100	92.5–100	
**Immunostaining**				
GLUT-1				
Positive	13 (44.8)	4 (22.2)	9 (81.8)	0.003
Negative	16 (55.2)	14 (77.8)	2 (18.2)	
HK-II				
Positive	10 (34.5)	2 (11.1)	8 (72.7)	0.001
Negative	19 (65.5)	16 (88.9)	3 (27.3)	
BRAF V600E				
Positive	10 (34.5)	0 (0.0)	10 (90.9)	< 0.001
Negative	19 (65.5)	18 (100)	1 (9.1)	
Nuclear expression of beta-catenin				
Observed	18 (62.1)	18 (100)	0 (0.0)	< 0.001
Not observed	11 (37.9)	0 (0.0)	11 (100)	

### Immunohistochemical study

GLUT-1 expression level was significantly higher in PCPs than ACPs (0, 1+, 2+, 3+ = 2, 12, 4, 0 cases in ACP, respectively, 0, 1+, 2+, 3+ = 0, 2, 5, 4 cases in PCP, p = 0.001, Mann–Whitney test) (Table 2), and most PCPs were classified into positive group (positive rate, 22.2% [4/18] in ACP, 81.8% [9/11] in PCP; p = 0.003, Fisher exact probability test) (Table 1). The expression of GLUT-1 was typically observed on epithelial cell membrane with a slight tendency to dominate expression in the basal cell layer. As well as GLUT-1, HK-II expression was also conspicuous in PCPs (0, 1+, 2+, 3+ = 7, 9, 2, 0 cases in ACP, 0, 3, 3, 5 cases in PCP; p = 0.001), and most of them divided into positive group (positive rate, 11.1% [2/18] in ACP, 72.7% [8/11] in PCP; p = 0.001) (Table 2). Expression pattern of HK-II showed no tendency like GLUT-1.

**Table 2:** Immunostaining score for anti-GLUT-1 and anti-HK-II.

Immunostaining grade	ACP, n = 18 (%)	PCP, n = 11 (%)	p value
**GLUT-1**			0.001
0	2 (11.1)	0 (0.0)	
1+	12 (66.7)	2 (18.2)	
2+	4 (22.2)	5 (45.5)	
3+	0 (0)	4 (36.4)	
**HK-II**			0.001
0	7 (38.9)	0 (0.0)	
1+	9 (50.0)	3 (27.3)	
2+	2 (11.1)	3 (27.3)	
3+	0 (0)	5 (45.5)	

Expression patterns of BRAF V600E and beta-catenin reflected the clinicopathological subtypes (BRAF V600E; positive rate, 0% [0/18] in ACP, 90.9% [10/11] in PCP, p < 0.001, nuclear expression of beta-catenin was observed, 100% [18/0] in ACP, 0%[0/11] in PCP, p < 0.001) (Table 1). One case of PCP, which was not detected BRAF V600E-positivity, showed low expression level of GLUT-1 and HK-II such as 1+ and 1+, respectively.

### FDG-PET

FDG-PET examination before surgery was possible in 2 ACP and 5 PCP cases. Individual SUVmax was 4.1 and 4.2 in ACP cases, and 9.3, 9.4, 10.7, 15.3, and 16.1 in PCP cases. FDG uptake was high in all 5 PCP cases with SUVmax > 9 (median, 10.7; IQR, 9.4–15.7).

### Representative cases

**Figure 2:**
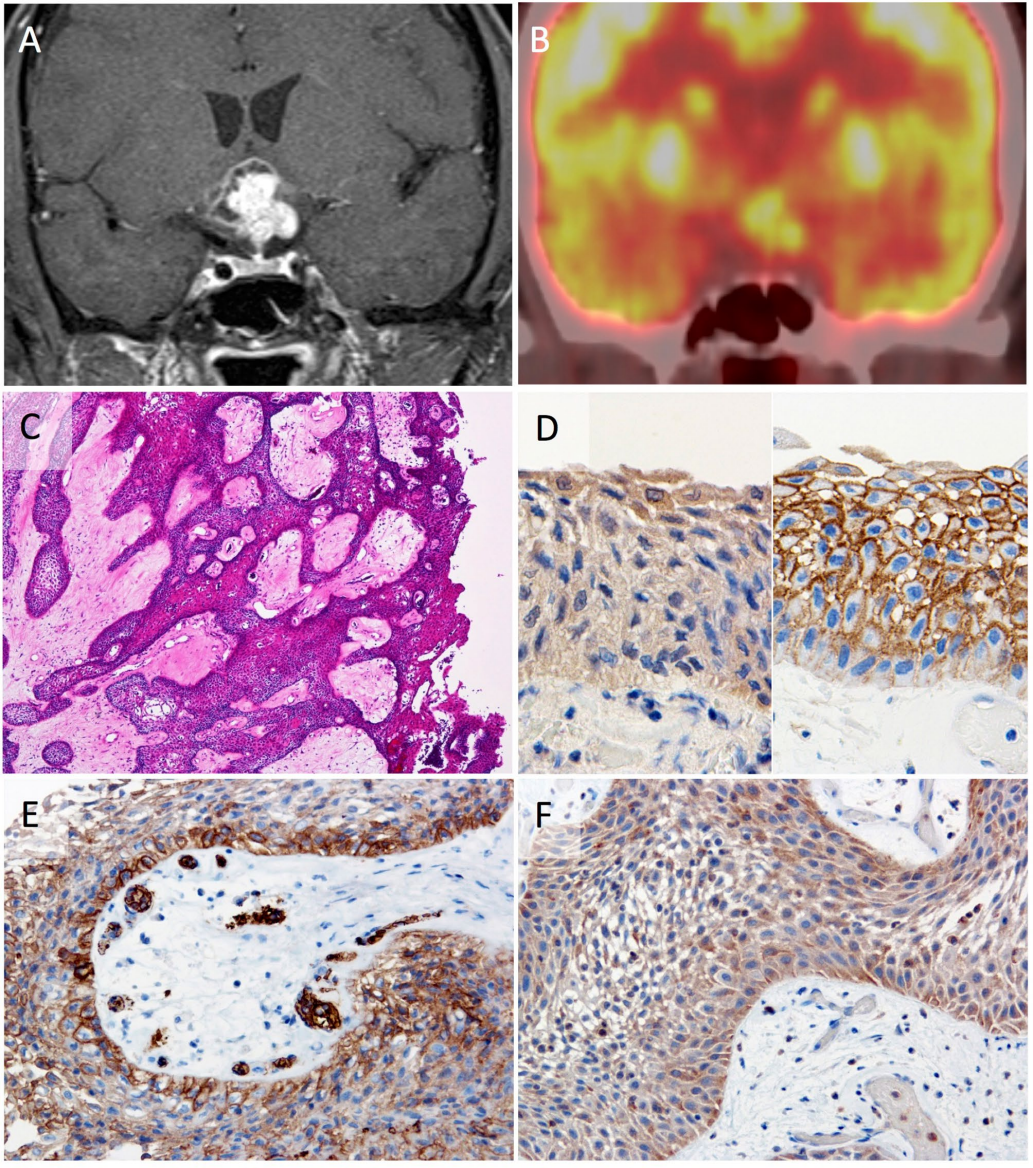
Case 1, a 57-year-old male patient with papillary craniopharyngioma. Preoperative coronal T1-weighted MR image after injection of gadolinium indicating an enhanced lesion with cysts in the suprasellar region (**A**). Preoperative [^18^F]fluorodeoxyglucose positron emission tomography (FDG-PET) coronal image demonstrating enhanced glucose uptake in the suprasellar tumor (**B**). Histologically, tumor shows papillary structures composed of non-keratinizing squamous epithelium with fibrovascular cores (**C**) (hematoxylin and eosin, original magnification ×40). The tumor cells are positive for BRAF V600E (**D**; left, ×400) and no expression of beta-catenin in their nuclei are observed (**D**; right, ×400). The tumor is markedly positive for GLUT-1 (**E**, ×200) and HK-II (**F**, ×200).

Case 1: A 57-year-old man developed headache, general fatigue, and visual field disturbance. Magnetic resonance (MR) imaging indicated a lesion with cysts in the suprasellar region (Fig. 2A). FDG-PET clearly showed enhanced glucose uptake in the solid part of the tumor with SUVmax of 9.3 (Fig. 2B). The tumor was removed through the endoscopic extended transsphenoidal approach. The histological diagnosis was PCP with the findings such as non-keratinizing squamous epithelium formed papillary structure (Fig. 2C). BRAF V600E and beta-catenin expression pattern sustained the diagnosis (Fig. 2D). Remarkable expressions were observed in both GLUT-1 (3+) and HK-II (3+) (Fig. 2E,F). Gross total removal was achieved. He remains in his job without recurrence for 4 years.

**Figure 3:**
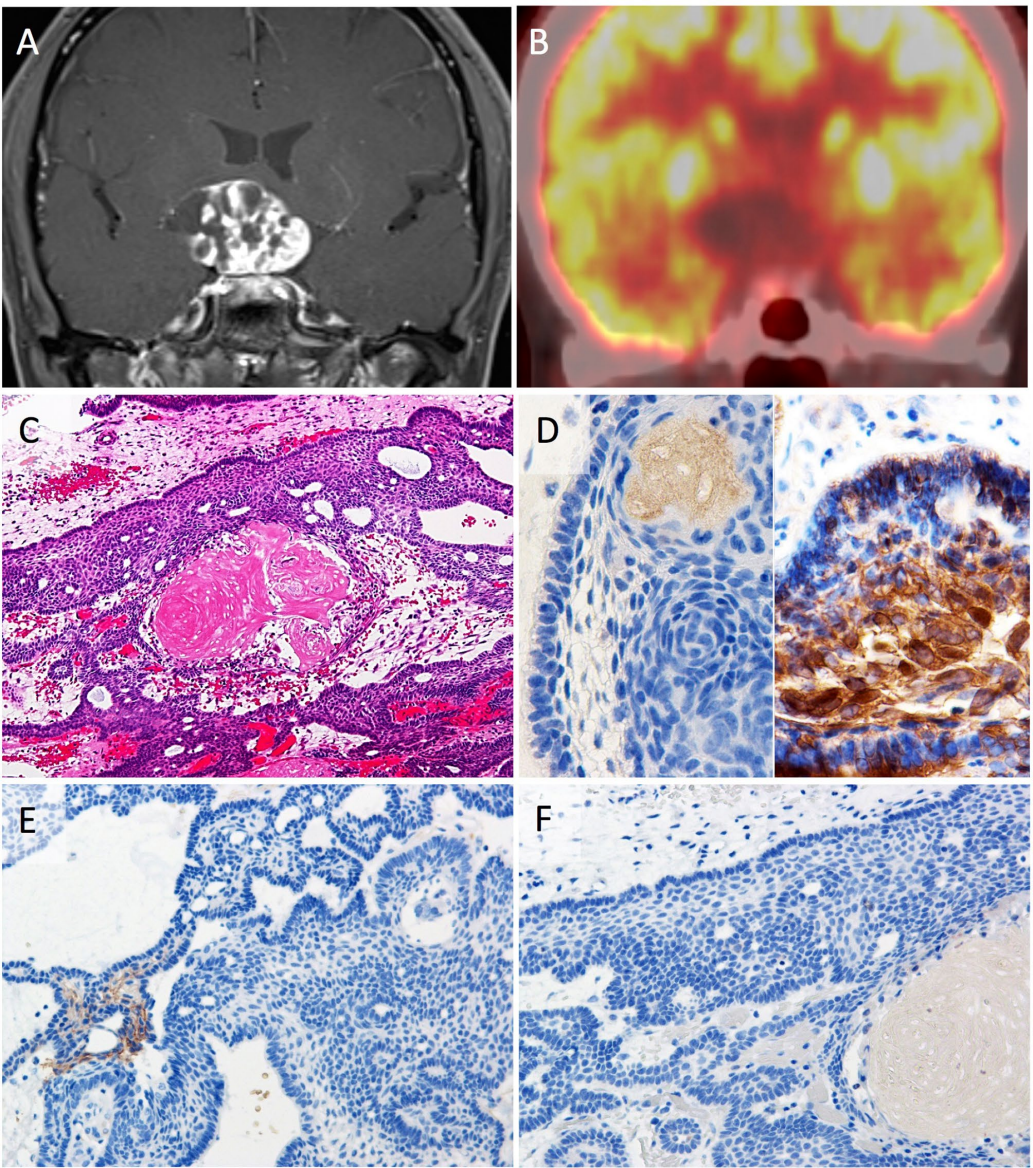
Case 2, a 39-year-old female patient with adamantinomatous craniopharyngioma. Preoperative coronal T1-weighted MR image after injection of gadolinium indicating a solid lesion with cysts in the suprasellar region (**A**). Preoperative FDG-PET coronal image demonstrating no glucose uptake in the suprasellar tumor (**B**). Pathologically, tumor with distinctive epithelium disposed in cords or lobules with basal palisading and wet keratin are detected (**C**) (hematoxylin and eosin, original magnification ×100). BRAF V600E is negative for tumor cells (**D**; left, ×400) and aberrant nuclear expression of beta-catenin is found (**D**; right, ×400). GLUT-1 expression is very focally detected (**E**, ×200). HX-II expression is not seen (**F**, ×200).

Case 2: A 38-year-old woman developed visual field disturbance. MR imaging indicated a solid lesion with cysts in the suprasellar region (Fig. 3A). FDG-PET did not show uptake in the solid part of the tumor with SUVmax of 4.1 (Fig. 3B). The tumor was removed through the endoscopic extended transsphenoidal approach. The resected tumor showed characteristic features of ACP such as stellate reticulum and wet keratin formed by well-differentiated epithelium (Fig. 3C). The results of BRAF V600E and beta-catenin immunostaining were corresponded to the diagnosis (Fig. 3B). Immunopositive area was very focal in GLUT-1 (1+) and no positivity was detected in HK-II (0) (Fig. 3E,F). The tumor was safely removed by more than 90%, and recurred after 6 months. She received second surgery with the same approach. The tumor was completely removed on postoperative MR imaging. However, very small recurrence was observed, and was treated by gamma knife radiosurgery. She remains in the same job without recurrence for 3 years.

## Discussion

This immunohistochemical study showed that expression of GLUT-1 and HK-II was common and strong in PCP, and rare and weak in ACP. Immunohistochemical staining for the BRAF V600E, and beta-catenin almost perfectly distinguished the clinicopathological variants. FDG-PET imaging showed very high uptakes of glucose with SUVmax over 9 only in several PCP cases.

The gain-of-function *BRAF* V600E mutation, a critical serine/threonine kinase in the RAS/RAF/MEK/ERK (MAPK) pathway, is a well established potent oncogene, so expression leads to increased cell proliferation and survival, resulting in cell transformation and tumorigenesis^11–13^. *BRAF* mutations have been found in melanoma (80–90%), thyroid cancer (60%), colorectal cancer (40%), non-small cell lung cancer (6%), pancreatic cancer (90%), and others^4–8,11–13^. Many tumors utilize the glucose metabolic system downstream of the *BRAF* V600E mutant protein. In human cancer cell lines, an increase in GLUT1 expression and glucose uptake was critically dependent on *KRAS* or *BRAF* mutations^4^. Mutations in *BRAF* cause cell proliferation through overexpression of GLUT-1 in papillary thyroid cancer^8^. *BRAF* and *KRAS* mutations have been correlated with GLUT-1 up-regulation in colorectal cancer^4,5^, and ovarian cancer^14^. Treatment of *BRAF* mutant melanoma with BRAF inhibitor reduced expression of GLUT-1, thereby suppressing tumor activity^15,16^.

A relationship between BRAF mutation and activation of MAPK downstream targets such as HIF1α and c-Myc and increased glucose metabolism were shown in melanoma^15^. HIF1α has been known to up-regulate expression of GLUT1 in hypoxic conditions. However, colorectal cancer cell lines with KRAS/BRAF mutations exhibited increased GLUT1 expression, independent of HIF1α status in normoxic conditions^4^. The mechanism between from BRAF mutation to up-regulates cellular glucose metabolism has not completely understood, but ERK regulates multiple transcription factors that control the expression of GLUT1^17,18^.

Clinically, evaluation of glucose metabolism depends on FDG-PET. Clinical studies have evaluated BRAF activity with FDG-PET. FDG-PET showed higher SUVmax in mutant *BRAF* than in the wild type of colorectal cancer^5^, and differentiated thyroid carcinoma^7^. The therapeutic effect of a BRAF inhibitor (vemurafenib) was evaluated by FDG-PET SUVmax in *BRAF* mutant melanoma^17^. Almost all PCP have the *BRAF* V600E mutation. On the other hand, ACP has the *CTNNB1* mutation, and these two genetic alteration are mutually exclusive^2,3,19^. Previous FDG-PET investigations of a series of sellar and parasellar tumors showed FDG uptake was high in PCP, but relatively low in ACP^10^.

Enhanced glucose metabolism in neoplastic lesions is associated with two independent systems, GLUT-1 and the glycolytic enzyme HK-II^5,9,20–22^. Tumor cells require excess glucose to maintain growth and proliferation. High GLUT-1 expression has been reported in various cancers, including colorectal cancer, lung cancer, breast cancer, ovarian cancer, melanomas, head and neck squamous cell carcinoma, and others^20^. The present study observed common and strong expression (2+, 3+) of GLUT-1 in 82% of PCP cases. In addition, GLUT-1 expression was found in 90% of BRAF V600E-positive craniopharyngioma cases (all PCP). In contrast, ACP showed no expression of BRAF V600E, and GLUT-1 expression was observed in only 22% of ACPs. Activation of the BRAF/MEK/ERK pathway in PCP may upregulate expression of the GLUT-1, and consequently affect glucose metabolism.

Hexokinase is an enzyme that catalyzes the first step of the glycolytic reaction. The type II isozyme is highly expressed in tumor cells^23^, and is thought to be the key enzyme in glucose metabolism and the Warburg effect^24^, Two steps are required to accumulate FDG in cancer cells: (1) facilitated diffusion through a glucose transport protein (GLUT-1); and (2) subsequent phosphorylation by one of the hexokinase isoforms (HK-II) to form FDG-6-phosphate. FDG-6-phosphate is not transported out of cells nor undergoes glycolytic breakdown^9,20–24^. HK-II expression is increased in many malignant tumors, including nasopharyngeal cancer, ovarian cancer, renal cell carcinoma, hepatocellular carcinoma, colon cancer, and glioma^20^. HK-II is related to the clinical stage, differentiation, metastasis, and poor prognosis of malignant tumors^9,21–24^. Many studies have focused on the correlation between GLUT-1 and HK-II activities and the FDG uptake in various types of tumors. Overexpression of GLUT-1 and HK-II is associated with enhanced tumor aggressiveness and poor survival^20–22,25–27^. However, HK-II expression is not completely consistent with GLUT-1 expression^4,5^. The present study showed common and strong expression (2+, 3+) of HK-II in 73% of PCP cases, and in 80% of BRAF V600E-positive craniopharyngioma cases (all PCP). In contrast, HK-II positivity was detected in only 11% of ACP cases. The signaling system from BRAF to HK-II remains unclear^15^. However, the present results suggest that HK-II acts to drive glucose metabolism in PCP, or *BRAF* mutated craniopharyngiomas.

BRAF inhibitors are highly effective against melanoma, non-small cell lung cancer, differentiated thyroid tumor, colorectal cancer, cholangiocarcinoma, and other tumors with the *BRAF* V600E mutation^28^. A case of PCP also showed remarkable response to a BRAF inhibitor^29,30^. So, identification of the clinicopathological variants of the tumor or the presence or absence of the *BRAF* mutation are necessary to optimize the chemotherapy regimen^28^. Craniopharyngioma is located in the deepest skull base region, and sample tissues are not easy to obtain (in contrast to melanoma). Therefore, imaging diagnosis of these clinicopathological variants is of great clinical importance^31,32^. Recently, a method was reported for diagnosing molecular variants (BRAF mutated or nonmutated craniopharyngioma) by preoperative MR imaging and CT^31,32^. However, this method depends on a combination of clinical and morphological phenotypes of tumors, which are essentially different from molecular imaging. Previous investigations of craniopharyngioma using FDG-PET showed strong uptake in PCP, but relatively low uptake in ACP^10^. The present immunohistological study revealed that *BRAF* V600E mutation-positive cases and GLUT-1- or HX-II-positive cases almost overlapped. FDG-PET can predict the effectiveness of BRAF inhibitor treatment and evaluate the subsequent effect. Recently, endoscopic extended transsphenoidal surgery without craniotomy has been widely introduced. In this series, 66% of cases were treated by endoscopic endonasal transsphenoidal surgery (Table 1). In cases of large intracranial tumor treated through a narrow nasal corridor, the extent of endoscopic transnasal tumor removal depends greatly on the size of the tumor^33^. Preoperative FDG-PET could be important in considering neoadjuvant BRAF inhibitor treatment strategy for craniopharyngioma in the future. Craniopharyngioma is a pathologically benign tumor classed as WHO grade I, and does not have specific carcinomatous properties such as metastasis. However, the presence of glucose-metabolizing activity with expression of GLUT-1 and HX-II as well as BRAF mutation may indicate potential malignant character.

The limitation of this study is the small number of cases collected over a long period and evaluated retrospectively. In particular, FDG-PET was performed for various reasons based on the clinical judgment of various neurosurgeons and physicians. Therefore, selection bias is present. Statistical analysis was not applicable to the small number of imaging results. Further prospective study is needed to establish the true diagnostic value of FDG-PET for subtype diagnosis of craniopharyngiomas.

## Conclusion

Both GLUT-1 and HK-II expressions were prominent in PCP. Glucose metabolism might be more enhanced in PCP than ACP. PCP may use the glucose metabolic system downstream of the BRAF V600E mutant protein. These new concepts may be useful in biological, pathological, and clinical considerations of craniopharyngiomas.
